# Fuzzy logic-based reactive power control for power factor enhancement in EV drives

**DOI:** 10.1038/s41598-025-34125-w

**Published:** 2026-01-16

**Authors:** Harshit Mohan, Arvind Kumar, Nitin Kumar Saxena, Shashank Mishra, Pavan Khetrapal, Brian Azzopardi, Vibhu Jately

**Affiliations:** 1 Department of Electrical and Electronics Engineering, University of Petroleum and Energy Studies, Dehradun, India; 2 Department of Electrical Engineering, Institute of Engineering and Technology, Lucknow, 226021 India; 3 Departmet of Electrical and Electronics Engineering, KIET Group of Institutions, Delhi-NCR, Ghaziabad, 201206 India; 4 Teerthanker Mahaveer University, Moradabad, India; 5 Department of Electrical and Computer Engineering, ABES Engineering College, Ghaziabad, India; 6 The Foundation for Innovation and Research–Malta, Birkirkara, BKR 4012 Malta; 7 MCAST Energy Research Group, Institute of Engineering and Transport, Malta College of Arts, Science and Technology (MCAST), Main Campus, Paola, PLA 9032 Malta; 8 Department of Systems and Control, Faculty of Engineering, University of Malta, Msida, MSD 2080 Malta; 9 Azzopardi & Associates, Birkirkara, BKR 2012 Malta

**Keywords:** Electric vehicle, Induction motor, Reactive power control, Fuzzy logic, Power factor, Energy science and technology, Engineering, Physics

## Abstract

Induction motors (IM) find broad applications in various Electric Vehicle (EV) drives because of their numerous advantages. The speed control of these EV motor drives is realized through different speed control algorithms such as direct torque control (DTC), field-oriented control (FOC), direct power control (DPC) etc. However, energy efficient control for these drives is still supplementary, especially in EV’s where drive cycle is irregular. This article contributes a fuzzy logic based reactive power control of IM drive for power factor enhancement, where the flow of reactive power is governed by reference speed and torque commands using fuzzy logic. The proposed methodology eliminates the necessity for integrating complicated flux estimators & observers and significantly improves power factor across a broad spectrum of EV drive cycle. To evaluate the performance and the effectiveness of the proposed technique, extensive simulations and experimental studies have been conducted on 0.75 kW induction motor drive considering the wide-ranging EV driving cycle and compared with the widely used FOC scheme.

## Introduction

Electric vehicles (EVs) can benefit from alternative fuels like hydrogen and biofuels, particularly in industries like heavy transportation where battery storage is less feasible. By combining these fuels with EV technology, future mobility systems will be more sustainable and energy sources will be more diversified. The induction motor (IM), particularly the squirrel cage type, ranks among the top candidates for Electric Vehicle (EV) propulsion applications (TeslaS-2014 & Toyota RAV4)^1–3^, because of its numerous advantages such as robustness, simple construction, low cost, and higher efficiency. Due to absence of permanent magnets, the manufacturing process of induction machine is considerably easier. Additionally, the multi-phase structure results in enhanced power density and reduced electromagnetic torque ripples^4–15^.

### Background and motivation

Several speed control schemes have been commercialized for high-impact automotive applications [EV and hybrid EV (HEV)], such as field-oriented control (FOC), direct torque control (DTC) and direct and indirect regulation of active and reactive power flows^9^. The field-oriented control technique facilitates torque and flux to be controlled in a decoupled manner mirroring the separately excited DC machine. Though the field-oriented control technique offers several advantages, however in sophisticated drive systems, where fine-tuned regulation is demanded, computation of precise flux is vital, moreover it is governed by machine-specific parameters. Several articles have been put forward to augment FOC’s operational stability for coping with parameters variability and uncertainties; however, these schemes introduce complexity and place greater computational strain on digital controllers^16–24^. The direct torque control is another widely used control scheme, which offers enhanced torque response—both static and dynamic through a predefined switching table and hysteresis-based control. In contrast with FOC, the DTC performs in a stationary reference frame and demonstrates lower responsiveness to parameter deviations and uncertain conditions; nevertheless, regarding high-performance drives, unpredictability of stator resistance changes, ineffective flux control, substantial torque ripple, and variable switching frequency grows unbearable. Incorporating vector-based modulation, filters and observers along with direct torque control scheme, makes it possible to tackle above issues to some extent, but at the cost of increased complexity and hence sacrificing the ease of conventional DTC scheme^25–31^. Furthermore, the advanced observer-based or model-predictive control strategies (super-twisting sliding mode with disturbance observer, nonlinear extended state observers combined with MPC, and observer-augmented MPC variants) that aim for high dynamic performance and robustness but require significant model knowledge, online estimation, or higher computational resources^32–34^. By contrast, our proposed fuzzy reactive-power control (FRC) targets a complementary design point: observer-free reactive-power driven flux adaptation for induction motor EV drives, with substantially lower implementation complexity and experimentally validated power-factor and RMS-current benefits on a practical dSPACE platform.

### Correlated work

The power-centered speed regulation strategy for IM has a very limited figure of publications^16^, and the offered methods are multifaceted. In recent times^17^, direct power control (DPC) and an indirect real and reactive power control (IRRPC)^18^ for the speed control of IM was proposed, where the decoupled current components are derived from reference active and reactive powers facilitating the independent regulation of torque and flux. The adoption of hysteresis controllers in conjunction with switching vector table degrades the operational performance due to ineffective flux control and pronounced torque ripple, and necessitating machine parameters-based coupling circuit. In DPC, the active and reactive power references are used to generate decoupled current components, however by using the look-up table, fixed states of power factor values only can be selected but not the intermediate one, which is very essential in high performance applications such as EV, where regular loading and unloading is performed. Furthermore, the reactive power reference generation is totally dependent on reference active power only but not on reference speed and reference torque independently, and hence the required power factor selection is not vibrant as change in reference active power remains same in two conditions when machine is loaded/unloaded with fixed reference speed or when machine reference speed is changed with fixed loading (Reference active power is the product of reference speed and reference torque). And hence makes this method unsuitable, particularly for EV applications.

### Innovation and organization

**Fig. 1:**
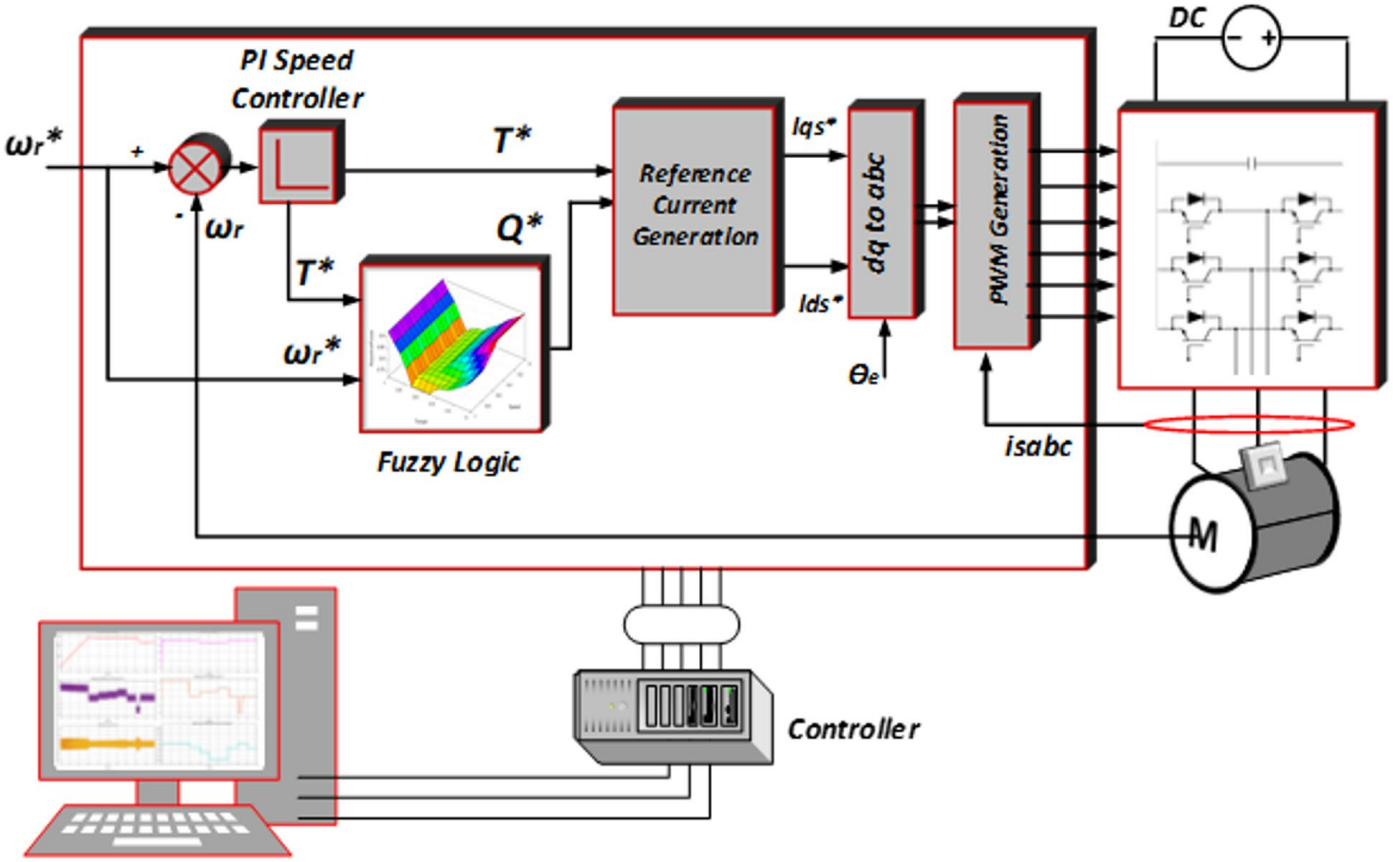
Block diagram of the reactive power-based speed control algorithm using fuzzy expert logic for induction motor drive.

The key features of the proposed work are given below:

- A reactive power-based speed control of induction machine using fuzzy expert logic is presented for electric vehicles. Figure 1 demonstrates the schematic circuit diagram of the proposed scheme. The reference speed and the actual speed are used to create reference torque, which further generates the torque producing component of current. The generated reference reactive power from reference speed and reference torque is used along with fuzzy expert logic to establish the flux-creating current component.
- In the proposed scheme, the estimation of flux is not mandatory, hence increases the robustness of the system. Along with this, the fuzzy expert system, which is quite effective for nonlinear applications, is used to decrease the system complexity such as intermediate transition states can be well selected without increasing the system dimension^19,20^ and it also disables the controller error.
- The proposed fuzzy expert logic is discussed thoroughly in this paper for EV drive cycle where the dynamic conditions are incorporated governed by reference speed and torque commands.

The rest of the paper is organized by discussing the EV dynamic analysis in section II, proposed control scheme using fuzzy decision-making system is elaborated in section III, MATLAB/Simulink validation for wide ranging EV cycle are shown in section IV, experimentally driven study is performed in section V, and concluding remarks with future direction of research is presented in section VI.

## Electric vehicle dynamics analysis

**Fig. 2:**
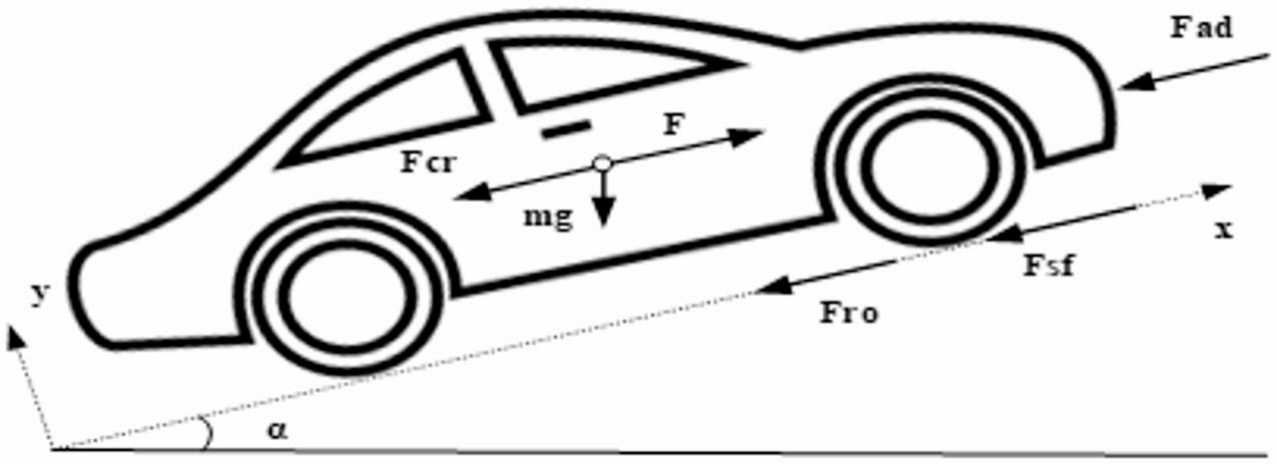
Fundamental forces acting on an Electric Vehicle.

Based on the directives of the vehicle mechanics and aerodynamics from literature study, the electric power and energy requirements for an electric vehicle can be evaluated by using the fundamental forces as shown in Fig. 2^2,3^.

where, *F* represents the tractive force in N, ‘*α’* is the inclination angle in rad, *m* is motor vehicle mass in Kg., *g* is gravitational acceleration in m/s^2^. The road load ‘*F*_*w*_’ or the tractive force comprises of resistive force resulting from tire flattening ‘*F*_*ro*_’, Stokes’ force or viscous friction force ‘*F*_*sf*_’, aerodynamic drag force *F*_*ad*_ due to air viscous resistance and climbing/downgrade resistance force *‘F*_*cr*_’ as;

1 $$\:{F}_{w}={F}_{ro}+{F}_{sf}+{F}_{ad}+{F}_{cr}$$,

2 $$\:{F}_{w}=\mu\:mgcos\alpha\:+{k}_{s}v+\raisebox{1ex}{$1$}\!\left/\:\!\raisebox{-1ex}{$2$}\right.\xi\:{C}_{AD}{A}_{f}{(v+{v}_{o})}^{2}\pm\:mgsin\alpha\:$$,

where, ‘*µ*’ is the coefficient of tire rolling resistance, ‘*k*_*s*_’ represents the Stokes’ coefficient, ‘*ξ’* is the air density, ‘*C*_*AD*_’ represents aerodynamic drag coefficient, ‘*A*_*f*_’ is the motor vehicle anterior area, ‘*v*_*0*_’ is wind velocity in m/s and *‘v*’ is the motor vehicle speed in m/s. Now, the equation of motion to overcome the road load by the EV can be mentioned as;

3 $$\:{k}_{r}mdv/dt=F-{F}_{w}$$,

where, ‘*k*_*r*_’ is rotational inertia constant. This net force is responsible for the acceleration or deceleration of the vehicle. Furthermore, motor rating and power transmission is designed by using the vehicle power required to offset the resistance from road load and derived as;

4 $$\:{P}_{v}=v{F}_{w}$$,

where, ‘*P*_*v*_’ is the motor vehicle driving power in Watts and from the mechanical equation of motor drive, the motor torque ‘*T*_*m*_’, consists of load torque ‘*T*_*L*_’, load torque accounting for friction & windage ‘*T*_*FW*_’ and inertial torque in N-m as;

5 $$\:{T}_{m}={T}_{FW}+{T}_{L}+Jd{\omega\:}_{m}/dt$$,

where, *‘J’* is the aggregate inertia of the rotor & the load in kg-m^2^ and ‘$\:{\omega\:}_{m}$’ denotes the motor’s mechanical rotational speed in rpm.

## Control algorithm

In this section the mathematical and logical approach for the reactive power-based speed control of induction machine using fuzzy expert logic is explained in particular and the corresponding nomenclature used are;

**Table:** 

Symbols	
*V* Voltage (*V*)	*σ* Magnetic Flux (Wb)
*I* Current (A)	*L* Self or magnetizing inductances (*H*)
*R* Winding resistance (Ohms)	*P* Number of pole pairs
Subscripts	
*d*,* q* Direct and quadrature axes	*s* Stator
*e* Electrical	*r* Rotor
*M* Magnetizing or mechanical	

### Computation of flux-inducing current component from reference reactive power

**Fig. 3:**
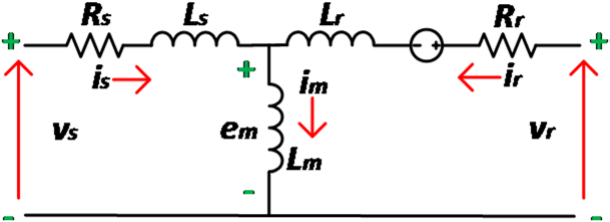
Per phase representation of 3-phase induction machine.

Real and reactive power are usually responsible for developing the electromagnetic torque and magnetic flux for the machine operation respectively. Since the magnetic excitation current is accountable for required flux establishment and hence employing the circuit diagram of induction machine from Fig. 3, the reactive power is computed as;

6 $$\:Q=\frac{3}{2}im\left\{{e}_{m}{i}_{m}^{*}\right\}$$,

where, $\:{i}_{m}=\frac{{\sigma\:}_{m}}{{L}_{m}}$. Owing to the fact that the stator leakage flux is comparatively insignificant to the magnetizing flux, it is supposed that $\:{\sigma\:}_{m}\approx\:{\sigma\:}_{s}$, and correspondingly the back emf is;

7 $$\:{e}_{m}\approx\:p{\sigma\:}_{s}\approx\:\frac{d}{dt}{\sigma\:}_{s}\approx\:j{\omega\:}_{e}{\sigma\:}_{s}$$,

where, $\:{\omega\:}_{e}$, is the source’s angular pulse frequency. Hence the Eq. (6) is expressible as a modified form;

8 $$\:Q=\frac{3}{2}im\left\{j{\omega\:}_{e}{\sigma\:}_{s}\frac{{\sigma\:}_{s}^{*}}{{L}_{m}}\right\}=\frac{3}{2}{\omega\:}_{e}\frac{{\left|{\sigma\:}_{s}\right|}^{2}}{{L}_{m}}$$,

Now since $\:{\sigma\:}_{s}\approx\:{\sigma\:}_{r}$, and the decoupling is accomplished through a technique akin to the vector control. The current component governing the flux production can be written as;

9 $$\:{i}_{ds}=(1+{\tau\:}_{r}p)\frac{{\sigma\:}_{s}}{{L}_{m}}$$,

From Eqs. (8) and (9), the d - axis current component is well-defined as;

10 $$\:{i}_{ds}=(1+{\tau\:}_{r}p)\sqrt{\frac{2}{3}\frac{Q}{{L}_{m}{\omega\:}_{e}}}$$,

Subsequently, the Eq. (10) quantifies the d – axis current component dictated by the specified reactive power.

### Fuzzy logic scheme

**Fig. 4:**
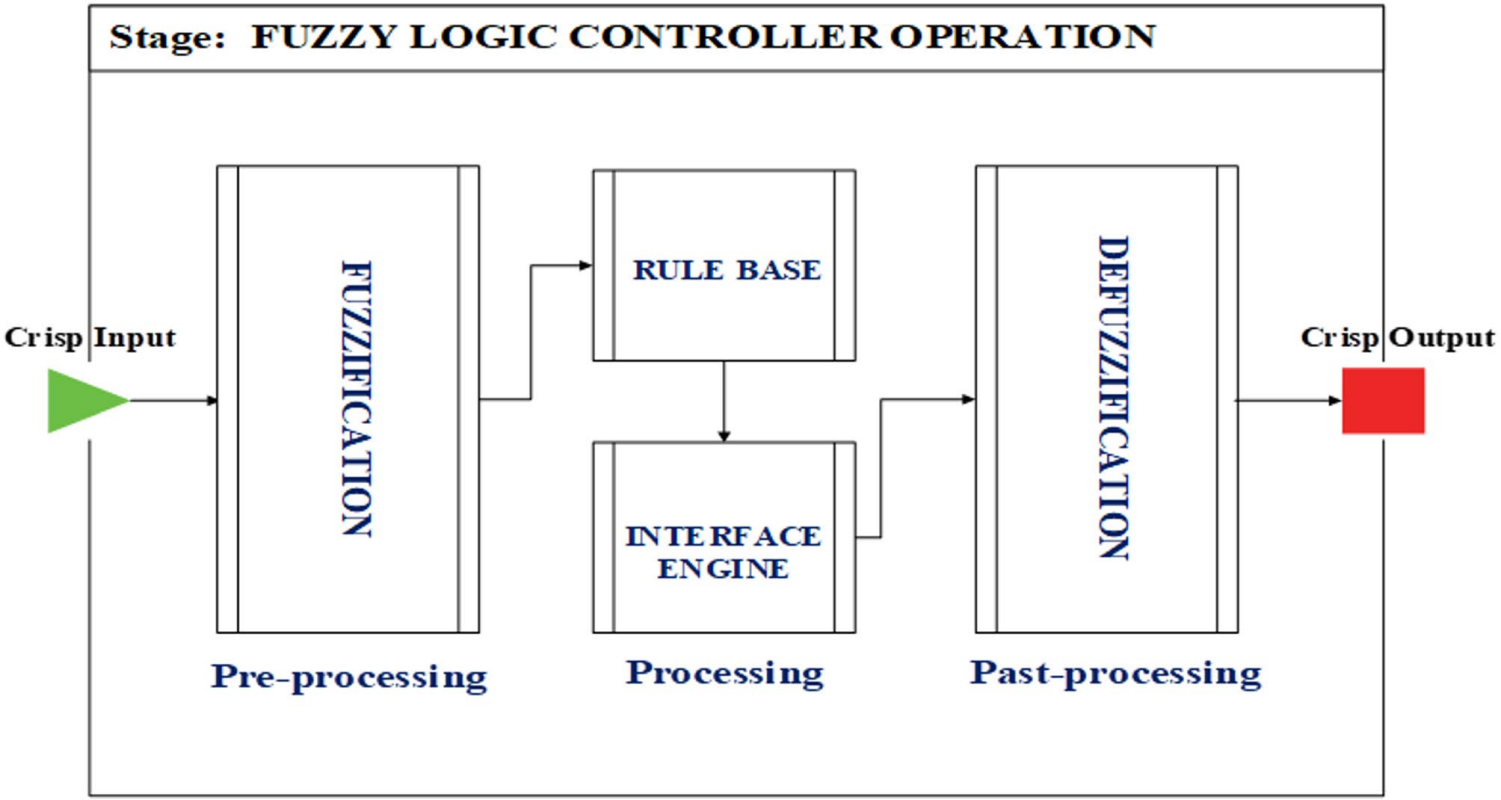
Operation stages of fuzzy logic controller.

Fuzzy rule – based system finds wide application for non-linear applications owing to its gain distribution functionality and saturation mitigation over an extensive operating range. The stages used for the fuzzy logic – based processing includes the input identification known as fuzzification, rule – based decision interface and precise execution of inference rules in producing crispy output known as defuzzification. Figure 4 illustrates the fundamental fuzzy logic framework.

**Fig. 5:**
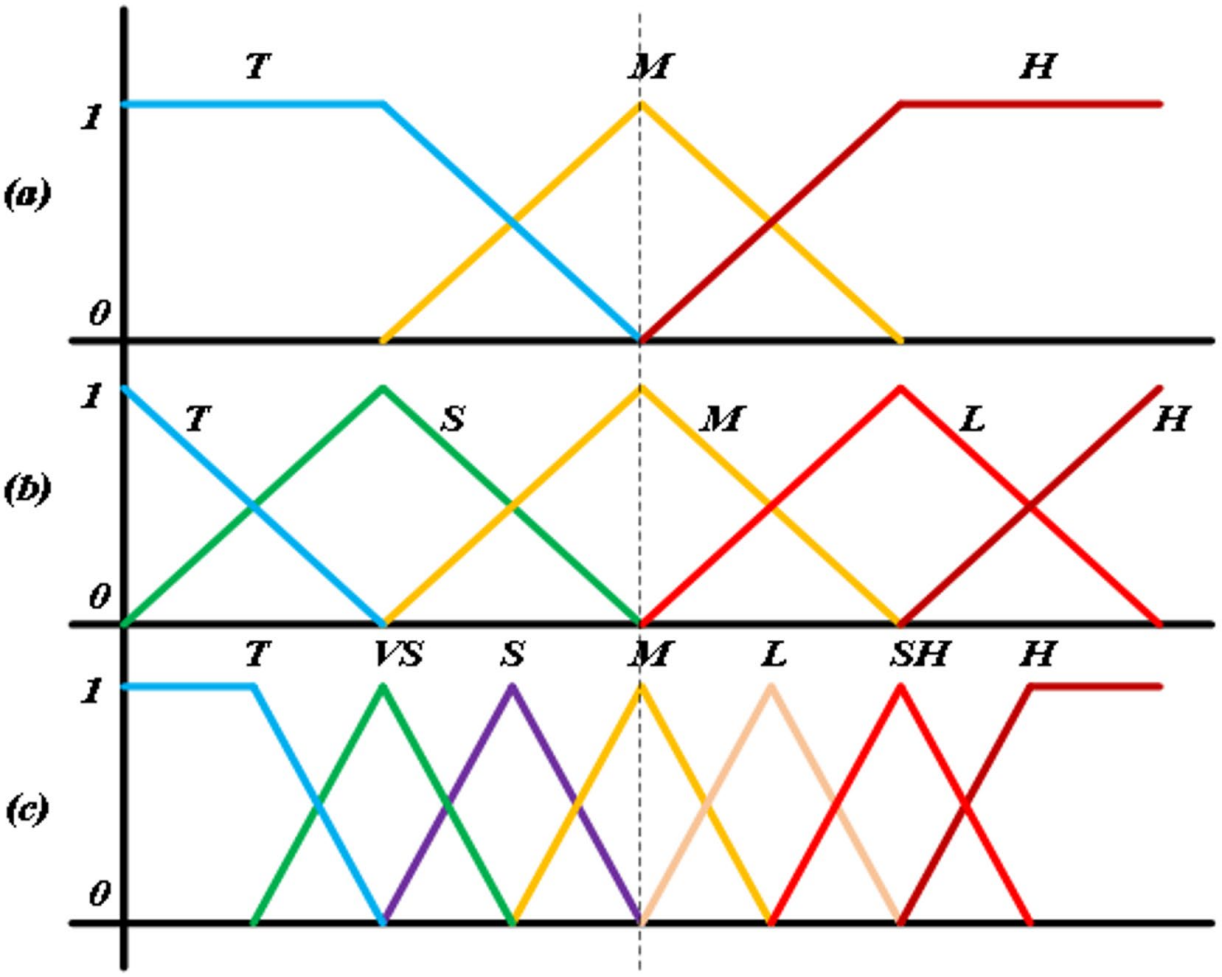
Various triangular MFs (**a**) 3 × 3, (**b**) 5 × 5 and (**c**) 7 × 7.

There are three types of fuzzy system; pure fuzzification system by Lotfi A. Zadeh^20^, Takagi-Sugeno (TS) fuzzification system by Takagi and Sugeno^21^ and Mamdani fuzzifier as developed by Ebrahim Mamdani^22^. The former one includes the pure fuzzy sets of input and output; however industrial systems have crisp values and hence cannot be implemented along with this. To overcome these constraints, Takagi-Sugeno fuzzification system was proposed, which consists of real crisp values of inputs and outputs. A simple mathematical formulation is used here to create the output, but again formulated output might not illustrate a conventional depiction of human artificial intelligence and it restricts to apply various values used in industrial applications. Hereafter, the Mamdani proposed the method which applies a fuzzifier that facilitates the conversion from crisp values to fuzzy equivalents and a defuzzifier to convert the fuzzy inputs into crisp counterparts^23,24^. Therefore, the various industrial machine processes are completed by using a Mamdani fuzzy rule controllers^25,26^ and so in this paper the Mamdani type fuzzy logic is used to implement on proposed technique. In fuzzy logic, the variable values may range from 0 to 1, contrary to conventional binary logic that only allows 0 or 1 value. The fuzzy values are linguistic values and defined as membership functions (MFs). Trapezoidal, Gaussian, Sigmoid, Triangular, and Singleton are the various types of MFs^27^. These MFs are normally chosen based on an inclusive understanding of the practical performance of the industrial system to be controlled. In fuzzification the various linguistic values are converted into fuzzy sets with suitable MFs. Different shapes and size of MFs are used in fuzzy logic controllers such as 3 × 3, 5 × 5 and 7 × 7 as shown in Fig. 5a–c, respectively. In addition, the scaling factors (SFs) are also significant parameters as it impacts overall system dynamic performance.

where, the various labels are; Tiny (T), Very Small (VS), Small (S), Medium (M), Low (L), Slight High (SH), and High (H). The fuzzy rule-base normally uses IF-THEN rule-base to express the correlation between various input and output variables using linguistic descriptors owing to its minimal complexity, wide usage, and computational efficiency. The total of these fuzzy rules is determined by the set of linguistic variables involved in the fuzzy inference process. Out of various rule-based designs, two methods are widely employed to build fuzzy rule base. The primary one, which is widely used was introduced by Mamdani, known as the Heuristic Method, which uses control engineering information and operative modeling and later one introduced by Takagi-Sugeno, referred to as the Deterministic Method, incorporates fuzzy modeling along with a self-adaptive fuzzy controller^28^. In Heuristic Method, the phase plane technique is widely used to connect rule-base with the time-response for improved dynamics, particularly for engineering drive applications^29–31^. The phase plane technique relies on expert system functionality without mathematical framework and here for close loop speed control induction machine drive, the rule set can be established as;

$$Rul{e_k}={\text{ }}If{\text{ }}e{\text{ }}is{\text{ }}{A_k}\;and\;De{\text{ }}is{\text{ }}{B_k}\;then\;DU{\text{ }}is{\text{ }}{C_k},$$

where, ‘*k*’ is the number of fuzzy rule, ‘*Rule*_*k*_’ is the kth fuzzy rule, ‘*e*’ and ‘*Δe*’ are the morphological variables of error and change in error with values ‘*A*_*k*_’ & ‘*B*_*k*_’ respectively, ‘*ΔU*’ is the output morphological variable with value ‘*C*_*k*_’. Here in this paper ‘*A’* and *‘B’* are respectively used for reference speed and reference load torque and ‘*C*’ is the corresponding reference reactive power output. This can be indicated by the equation shown below;

11 $$\:Fuzzy\:logic\:output\:Q\:=\:function\:(Reference\:Speed,\:Reference\:Load\:Torque)$$

Although the selection of larger size MFs could improve the industrial system efficiency but potentially increases the system’s computational processing requirements. And hence in this paper a 5 × 5 size of MFs are used. The different sets of rules used are shown in Table 1 below, where the different levels have their common denotations defined earlier.

**Fig. 6:**
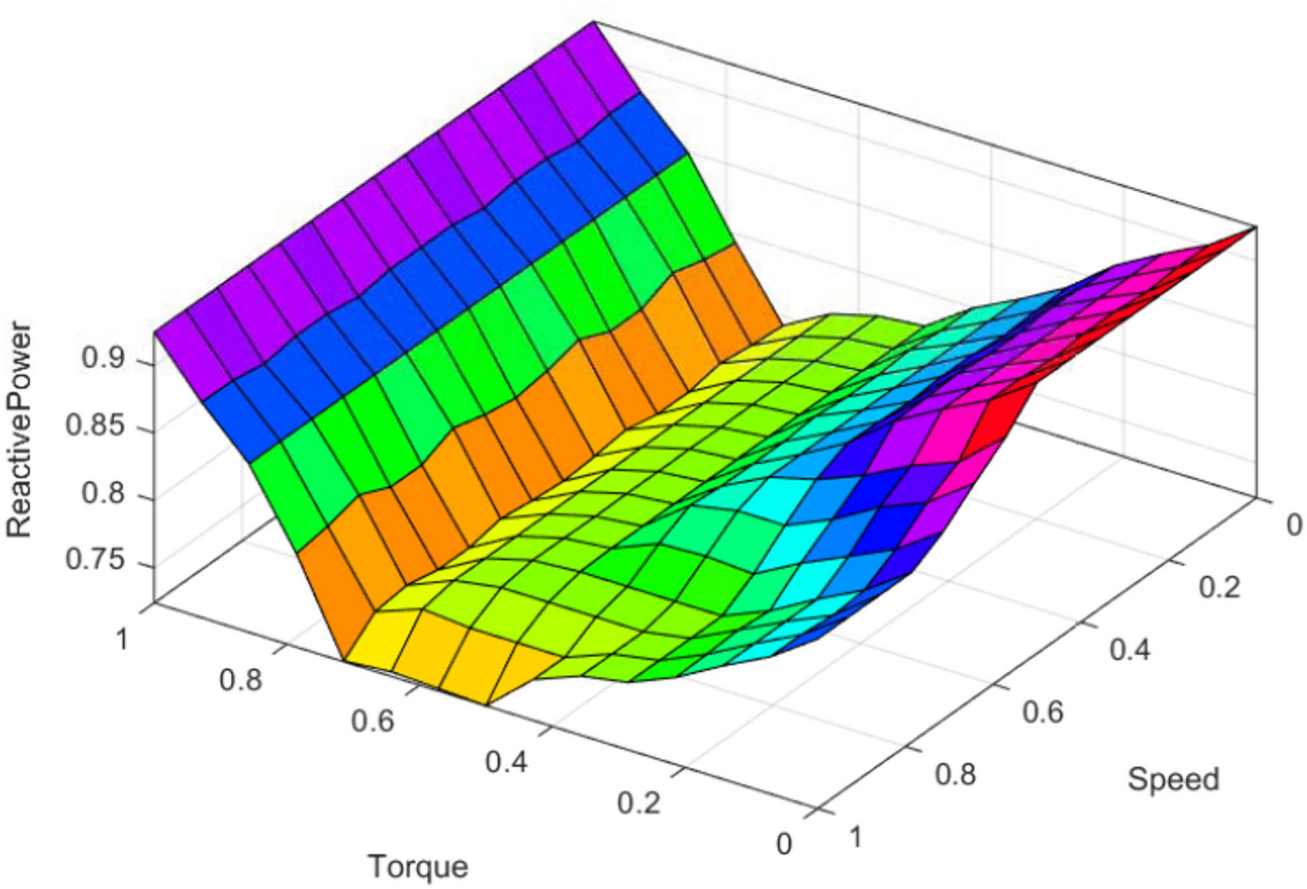
Surface rule diagram of the proposed control scheme.

Table 1 shows the rules used for the proposed scheme where the input set comprises torque and speed and depending upon various conditions of loading and variable speed, the reactive power output rules are defined such that improved power factor is attained. Here the various labels Tiny (*T*), Small (*S*), Medium (*M*), Low (*L*), and High (*H*) and have quantification value 75%-80%, 80%-85%, 85%-90%, 90%-95% and 95%–100% respectively in the permissible range. To describe the centroid of the two-dimensional functions the Mamdani defuzzification method is selected. The three dimensional surface rule diagram corresponding to the Table-I for the proposed scheme is shown in Fig. 6. In three dimensional surface rule diagram, the per unit speed and load torque are used as the inputs in x and y axis respectively and reactive power as the output in z axis.

**Table 1:** Fuzzy control rules.

Torque	Rule				
	Speed				
	T	S	M	L	H
T	L	L	M	M	M
S	M	M	M	S	S
M	S	S	S	S	T
L	T	T	T	T	T
H	L	L	L	L	L

The rule derivation aligns with the physical relationship between torque, speed, flux, and reactive power as;

- Higher reference torque demands higher flux only during heavy loading.
- Higher reference speed increases back-EMF and reduces required magnetizing current.
- This creates a nonlinear surface relating speed–torque–reactive power, which the fuzzy rule base approximates.

Further, phase-plane reasoning is applied, following classical fuzzy-control literature (Uddin, Masiala, Mamdani rules).

- Reference speed (input 1) and reference torque (input 2) form the state plane.
- Reactive-power command (output) is selected to achieve minimal flux for each operating quadrant while maintaining stability.

SFs are applied to normalize:

- input variables (speed error, torque command);
- the fuzzy output (reactive power);
- and the final flux-producing current i_d.

This ensures that the fuzzy inference operates within a well-defined universe of discourse.

In the fuzzy logic controller, these calibrated per unit signals of torque and speed are introduced and corresponding to which crisp output is obtained and calibrated accordingly to calculate the current component that induces the flux as validated in the next section.

## Simulation analysis

In this section, the performance of the proposed fuzzy expert-based speed control scheme of EV induction motor drive using reactive power is simulated in the MATLAB/Simulink 2018b platform. The parameters used to simulate the proposed scheme on 0.75 kW machines are itemized in Table 2. The efficacy of the decoupled and precise control of the proposed technique is compared with the widely used vector control technique that is analyzed for different case studies.

### Case study 1: operation under variable speed and load scenarios

**Fig. 7:**
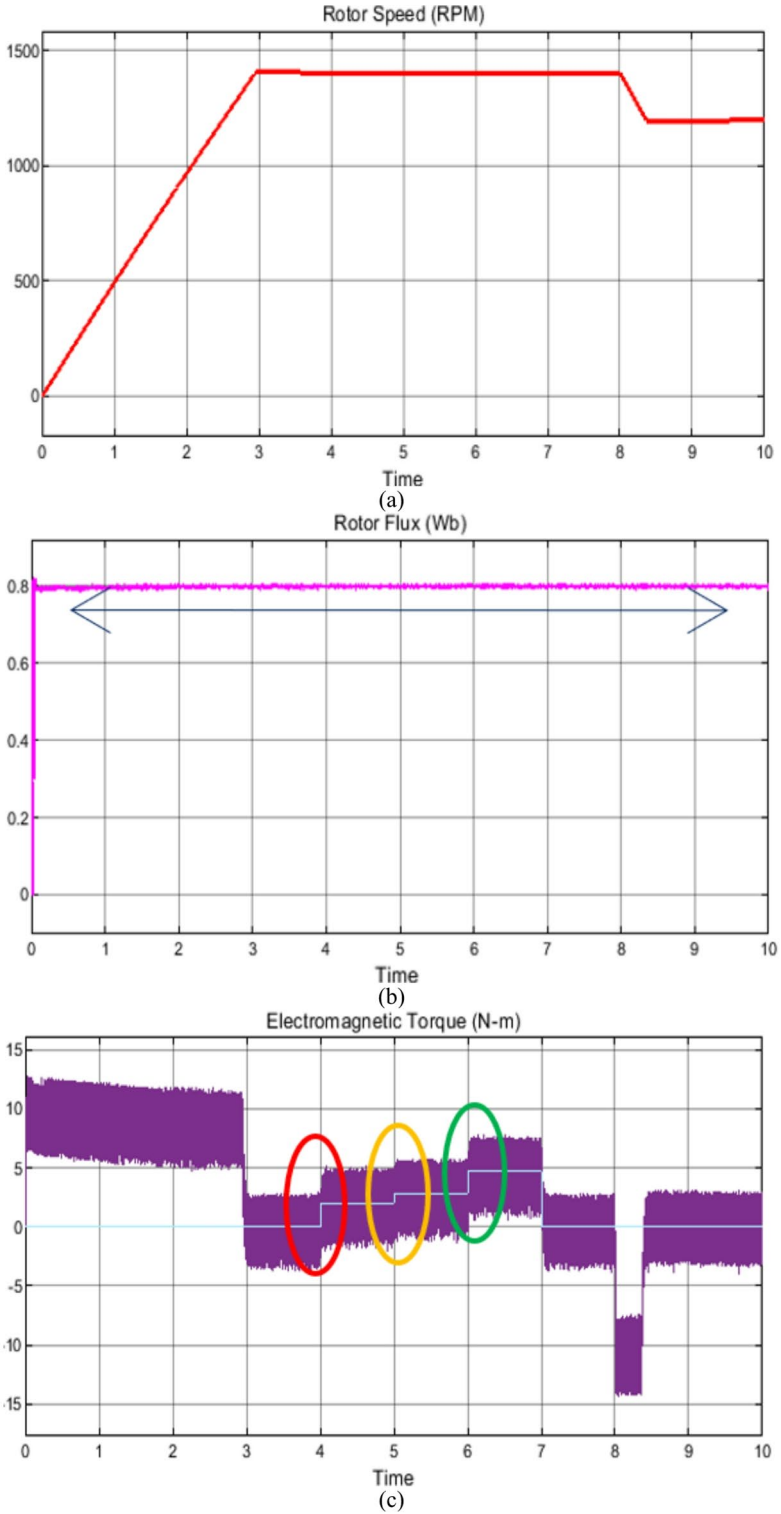

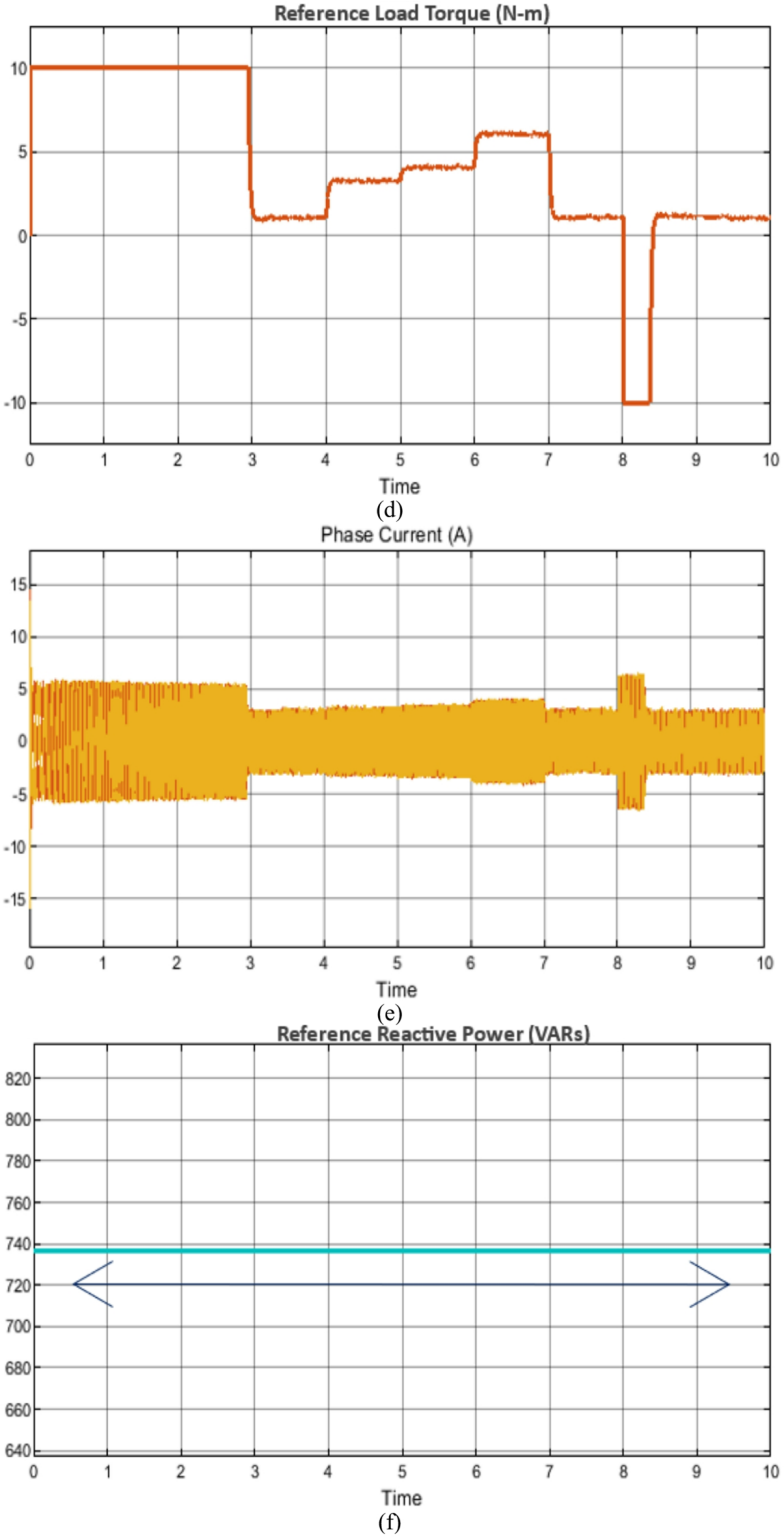
Simulation results of the state-of-the-art vector control technique under wide range of operating conditions (**a**) rotor speed, (**b**) rotor flux, (**c**) Electromagnetic torque, (**d**) Reference load torque, (**e**) Phase current and (**f**) reference reactive power.

**Fig. 8:**
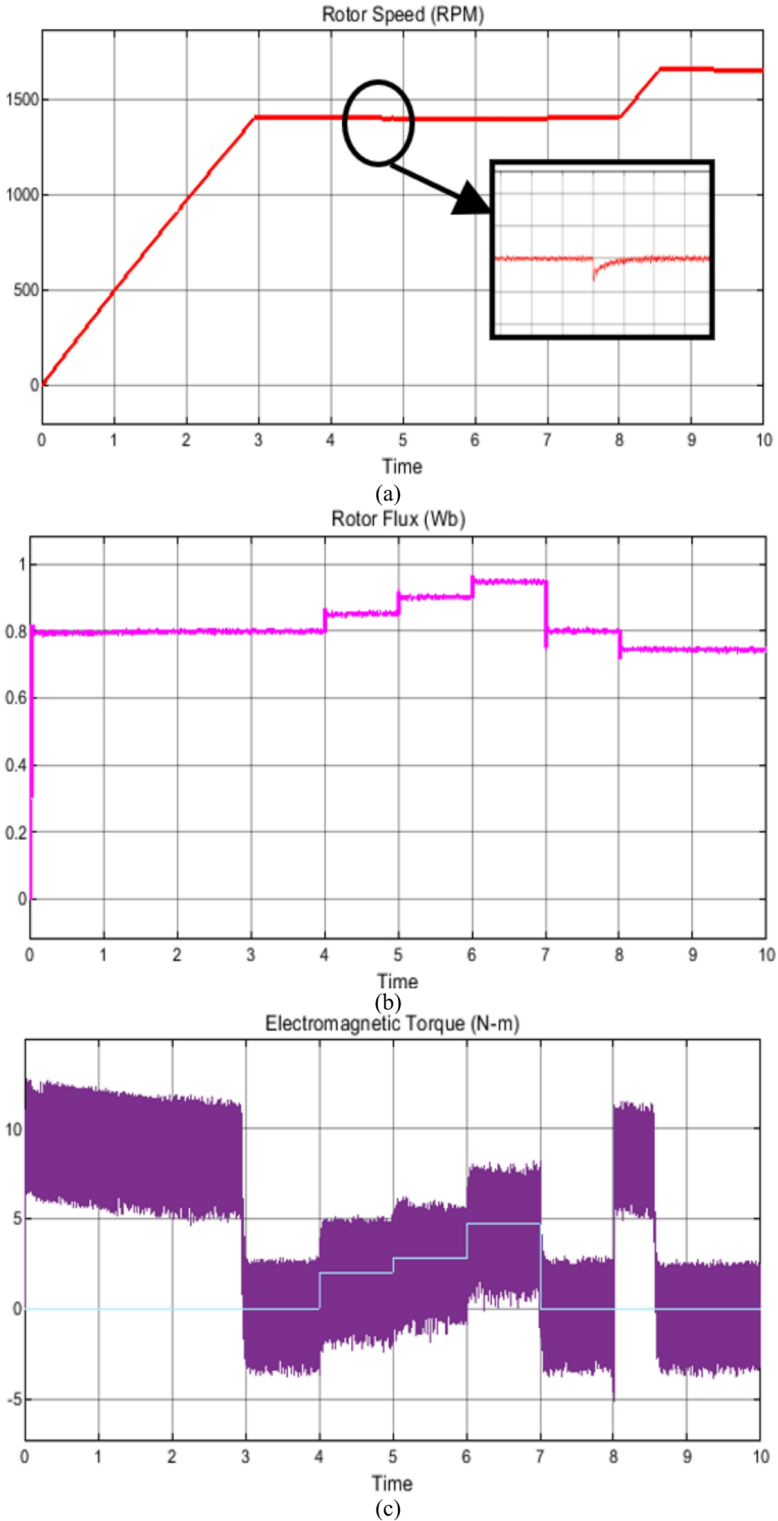

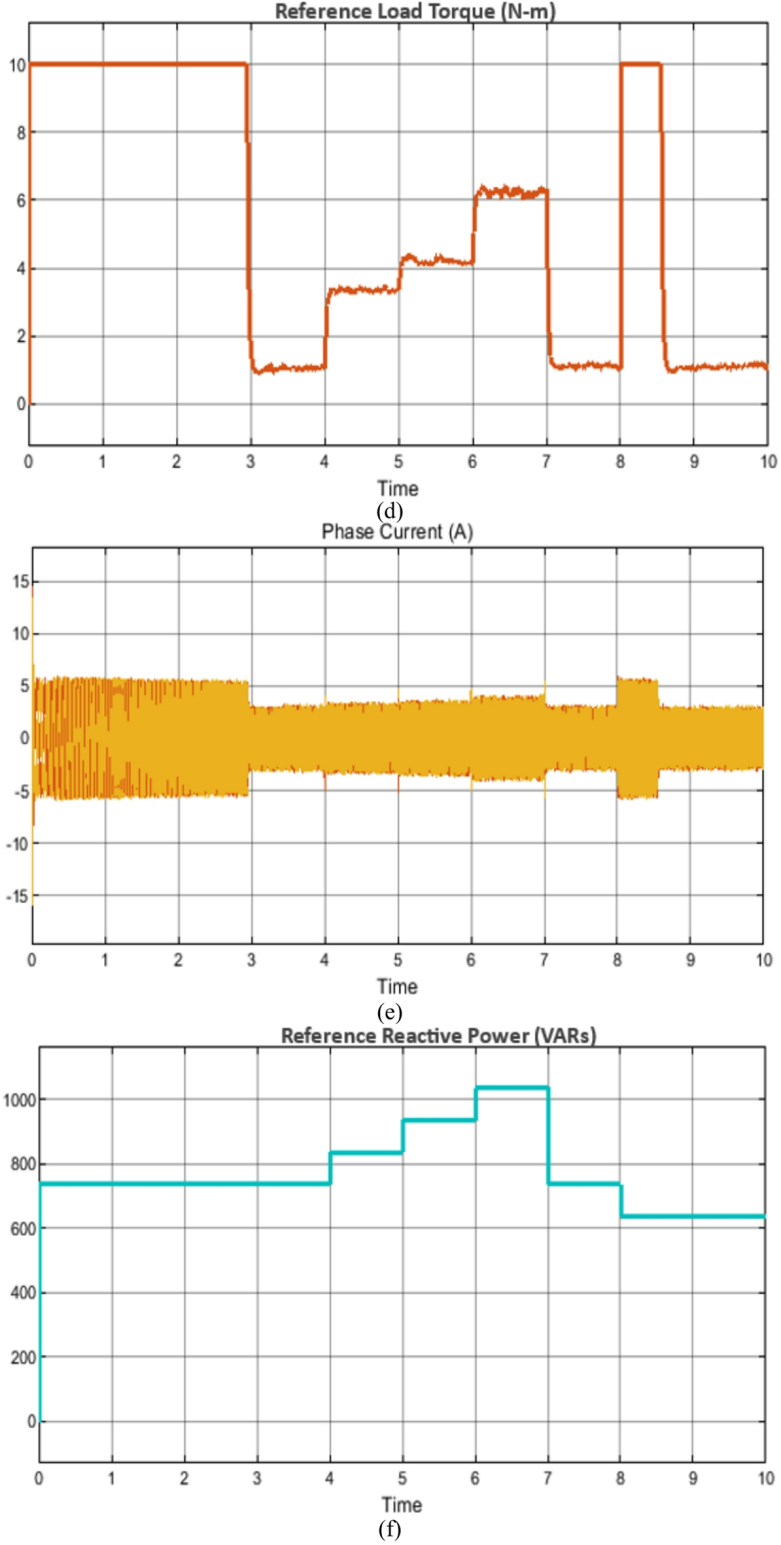
Simulation results of the proposed control technique under wide range of operating conditions (**a**) rotor speed, (**b**) rotor flux, (**c**) Electromagnetic torque, (**d**) Reference load torque, (**e**) Phase current and (**f**) reference reactive power.

The behavior of the vector control technique and proposed scheme under variable speed and load is shown in Figs. 7 and 8, respectively. Initially, the soft starting up to reference speed of 1400RPM is achieved and thereafter the machine is subjected to increasing load from unloaded operation to full load condition respectively as shown in Figs. 7 and 8. The corresponding plots of phase current, rotor flux, reference load torque and fuzzy output of reference reactive power are also shown for the state-of-the-art vector control and the proposed control technique in Figs. 7 and 8, respectively.

**Table 2:** 0.75 kW induction machine parameters.

Parameters	Values	Parameters	Values
Power	0.75 kW	Rotor Resistance	8.9838 ohms
Voltage	415 V, (L-L)	Leakage Inductance	0.0336 H
Frequency	50 Hz	Coupling Inductance	0.49051 H
Pole	4	Inertia	0.00181 kg- m^2^
Stator resistance	11.124 ohms	Rated Speed	1500 RPM

**Fig. 9:**
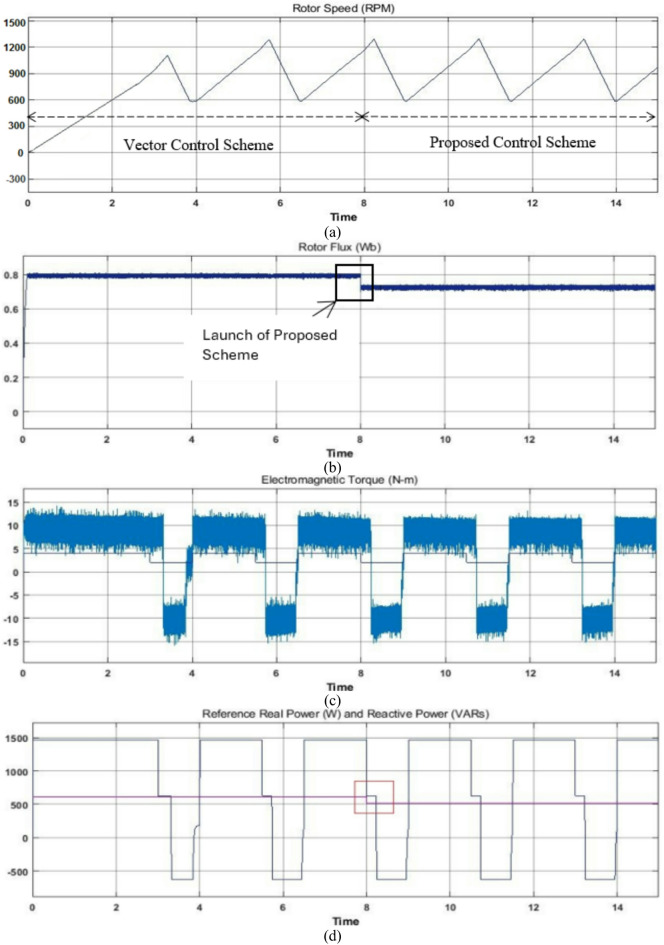

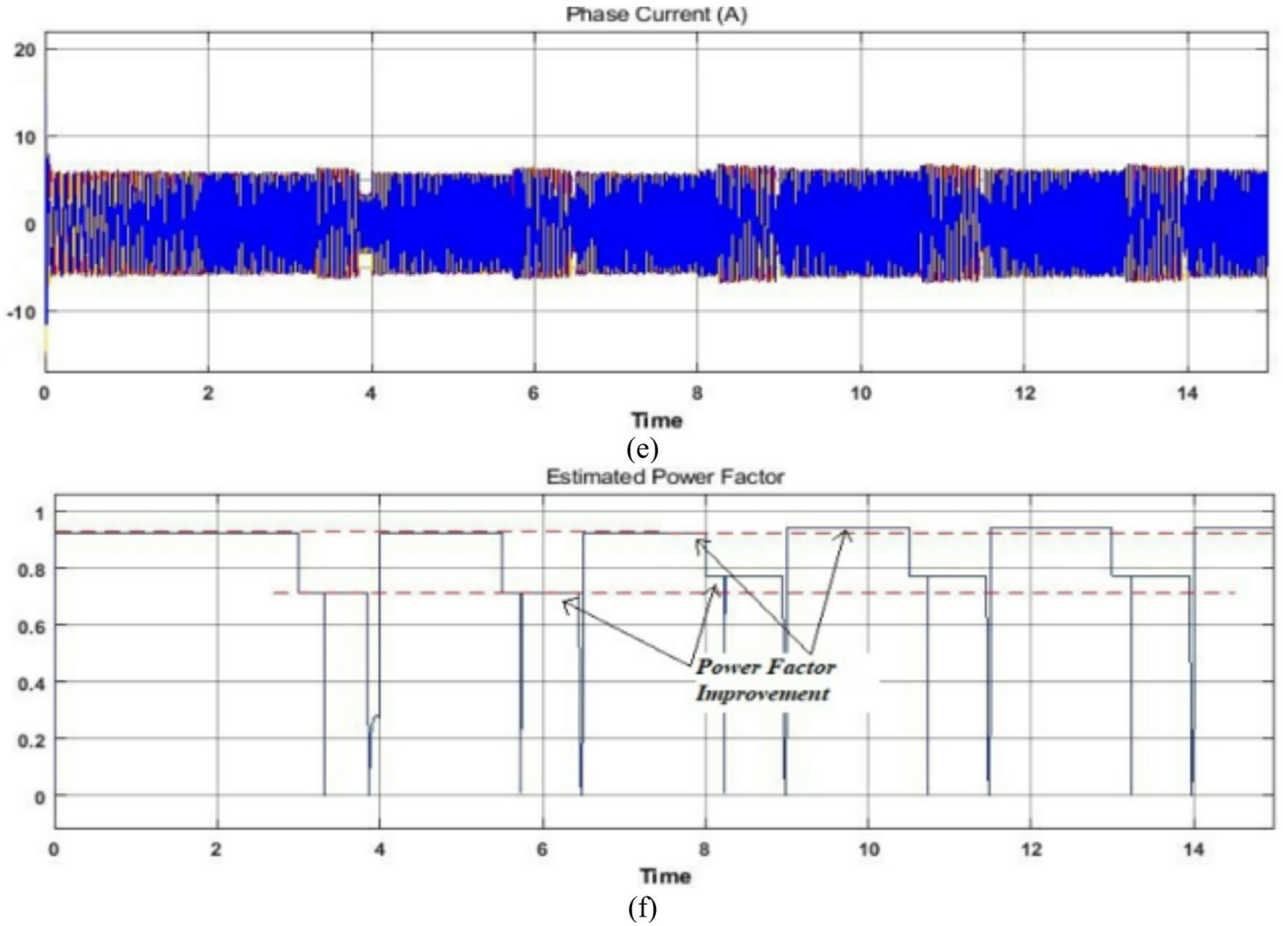
Simulation based performance analysis for the vector and the proposed control scheme on a variable load tested for a wide range of drive cycle.

**Fig. 10:**
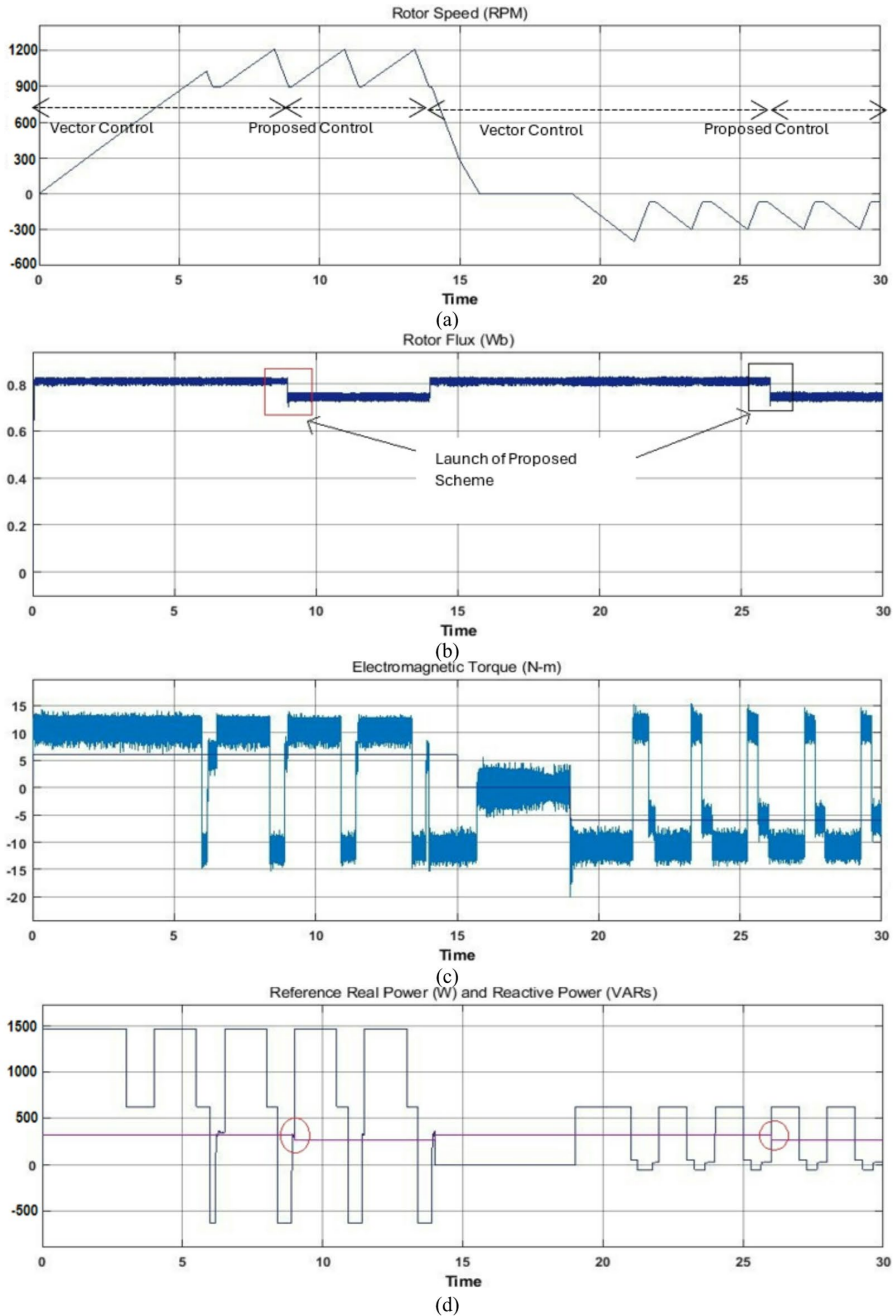

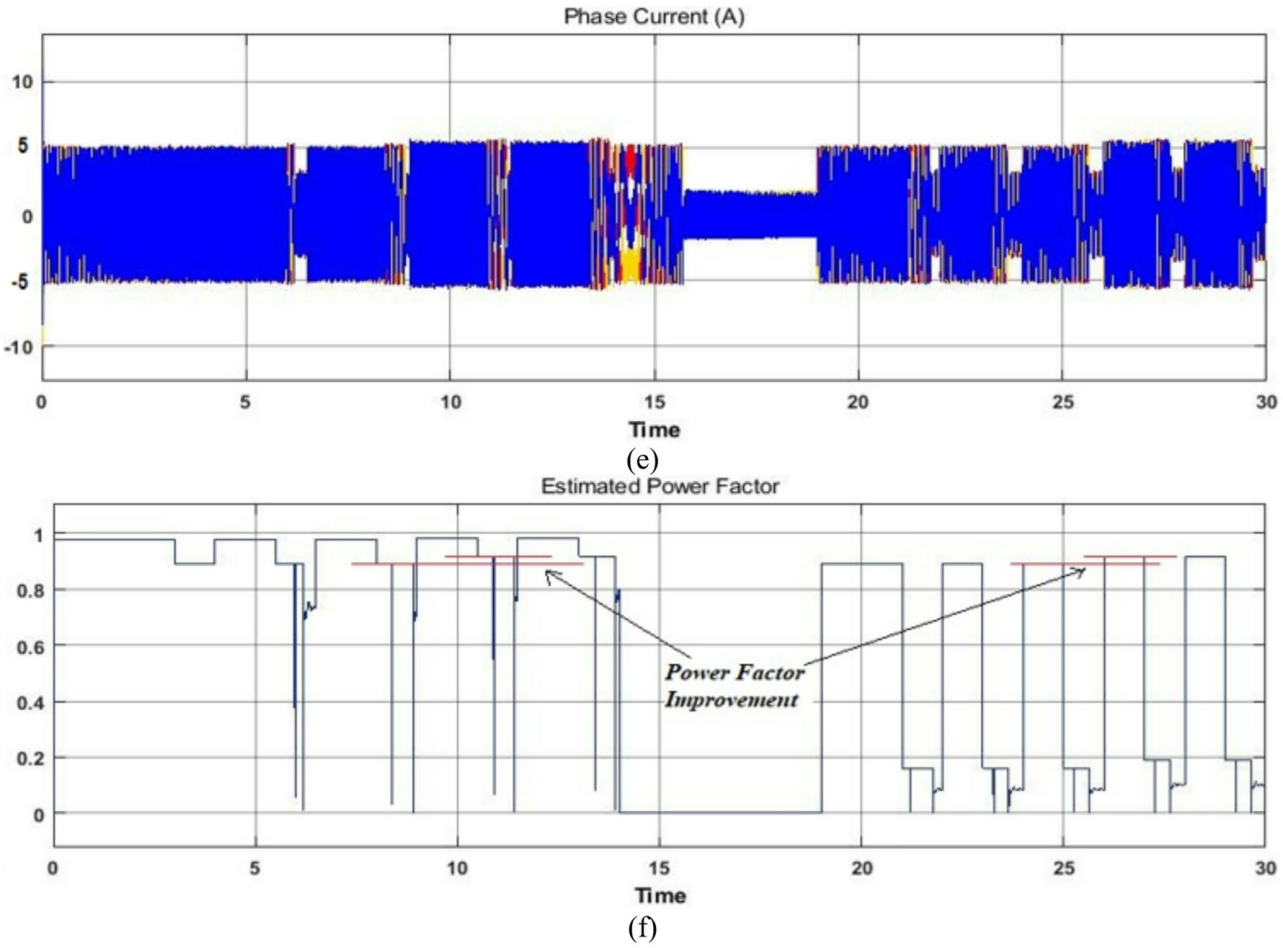
Simulation based performance analysis for the vector and proposed control scheme on a fixed load tested for a wide range of drive cycle.

Nevertheless, in this operation the depending upon the loading and desired speed, the fuzzy controller positively creates the reference reactive power output which correspondingly changes the flux with precise and decoupled speed control. In comparison with the existing vector control scheme, where the operating flux remains constant irrespective of variation in load torque and/or speed, the proposed scheme requires reduced reactive power which increases the operating power factor.

### Case study 2: operation under variable and fixed load for a wide range of drive cycle

To additionally demonstrate the outperformance of the proposed controller it is subjected to testing over a wide range of drive cycles to validate the proposed scheme for EV application. The selection of driving cycles plays a significant role in analysis and design of propulsion systems, since driving patterns have a stringent impact on specific energy consumption^6–9^. For the purpose of emulating different road conditions, a driving cycle is considered with variable loading as shown in Fig. 9. This driving cycle is similar to the motor vehicle driving on a descending slope. The corresponding electromagnetic torque, phase current, rotor flux, reference real & reactive power and estimated power factor plots are also displayed for the justifications of the proposed control scheme. To demonstrate the validations and comparison of the proposed scheme with other control technique (particularly the widely used vector control scheme), until time t = 8s, the motor is operated with the vector control scheme. At time t = 8s, the proposed reactive power control technique is applied with fuzzy logic controller. During the complete time duration motor achieves continuous operation of required driving cycle impeccably as shown by rotor speed, electromagnetic torque and phase current plots. However, the differences are obtained only in rotor flux and reference reactive power plots as shown. In estimated power factor plot of Fig. 9, the two parallel red dashes lines are shown, which compares the estimated power factor of the vector control and proposed control scheme. The upper one compares the high power factor values and lower one compares the low power factor values of the driving EV driving cycle. After t = 8s, the upper as well as the lower power factor values are higher than the values before t = 8s of the vector control scheme as shown in Fig. 9. And hence improved power factor is obtained for wide range of driving cycle without deteriorating the performance.

Another driving cycle is considered under constant load during both motoring and reverse motoring conditions as illustrated in Fig. 10. This driving cycle is analogous to the motor vehicle driving on a level road. Here also, until time t = 8s, during motoring mode with constant load vector control operation is employed. The corresponding electromagnetic torque, phase current, rotor flux, reference real and reactive power and estimated power factor plots are displayed for this mode of operation to justify the proposed control scheme. At time t = 8s, the proposed reactive power control technique is applied with fuzzy logic controller and respectively the rotor flux and reference reactive power changes to improve the power factor as shown (Noticeable in red parallel lines). After some time under same operating conditions the reverse motoring mode is performed with vector control scheme at initial stage from time t = 15s to t = 26s and later at time t = 26s, the same action of the proposed scheme is carried out during reverse motoring mode and here as well the improved power factor is achieved as shown in Fig. 10. Hence the simulation studies of Figs. 9 and 10 confirm the suitability of the proposed algorithm during motoring, reverse motoring, fixed and variable loading conditions. In the subsequent section experiments are carried out to analyze the performance of the proposed scheme for real-world scenarios.

## Experimental analysis

**Fig. 11:**
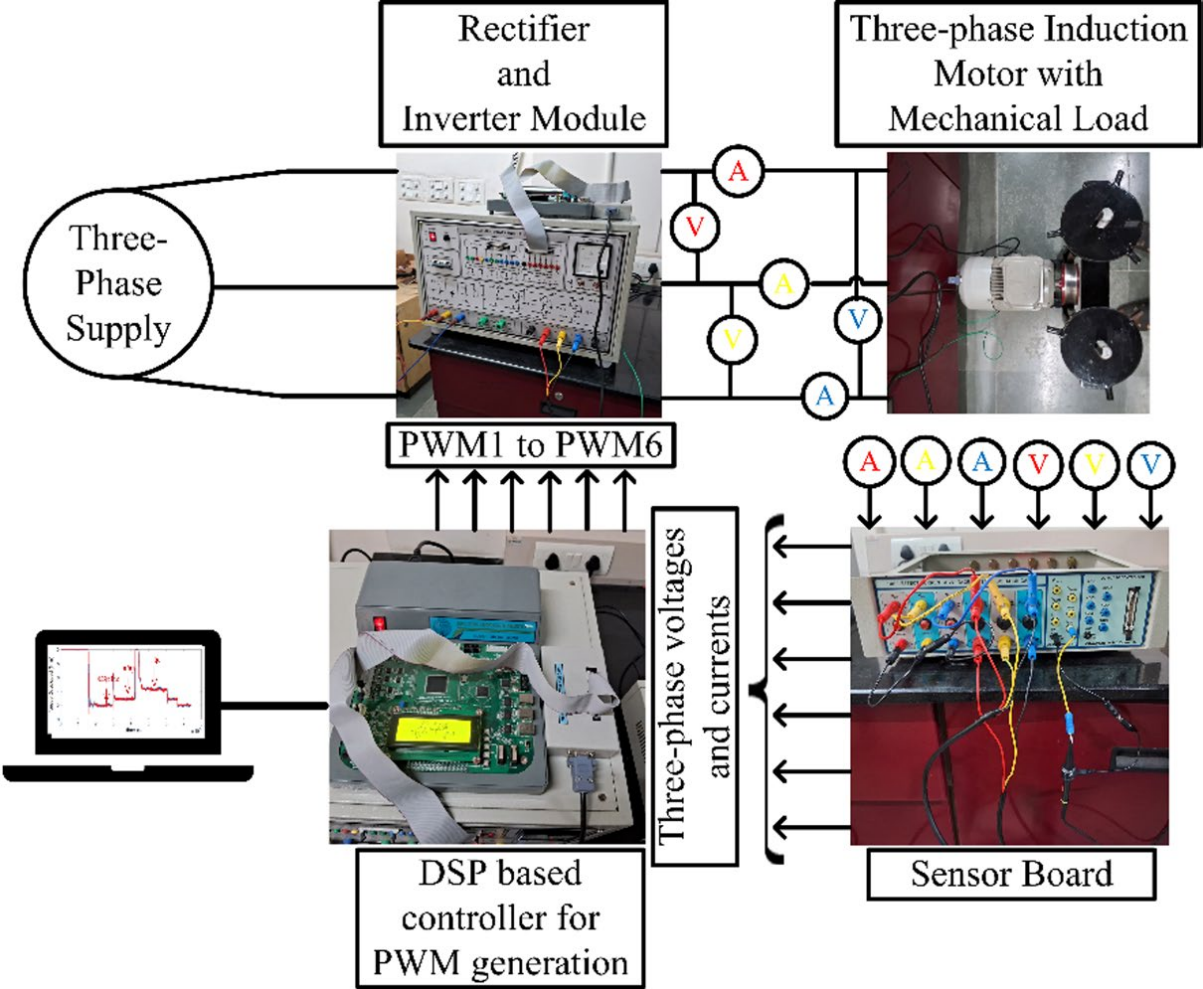
Lab-scale experimental arrangement for 0.75 kW induction machine.

To validate the proposed algorithm experimentally, a hardware setup is developed as depicted in Fig. 11. The test configuration is made up of 0.75 kW induction machine integrated with a 0.75 kW self-excited direct current generator powering the bulb load, respectively marked in the photograph. The DSP centric dSPACE-1104 functions as controller to generate PWM pulses of variable switching frequency with a maximum of 5 kHz for the 2-level IGBT inverter module with sampling time of 100e^− 6^ seconds. The 415 V, 50 Hz inverter module consists of a diode rectifier and an IGBT inverter module with DC Link of 600 V. The dSPACE Control Desk, Fluke Power Quality Analyzer and 4-Channel Digital Signal Oscilloscope are implemented to capture real time operating conditions of the different variables including the power factor.

To justify the decoupled speed and/or torque control of the proposed scheme, lab-scale experimental case studies are carried out similar to the simulation analysis carried out using MATLAB/Simulink as the simulation platform and the empirical findings are compared with the vector control scheme.

### Case study 1: operation under variable speed and load conditions

**Fig. 12:**
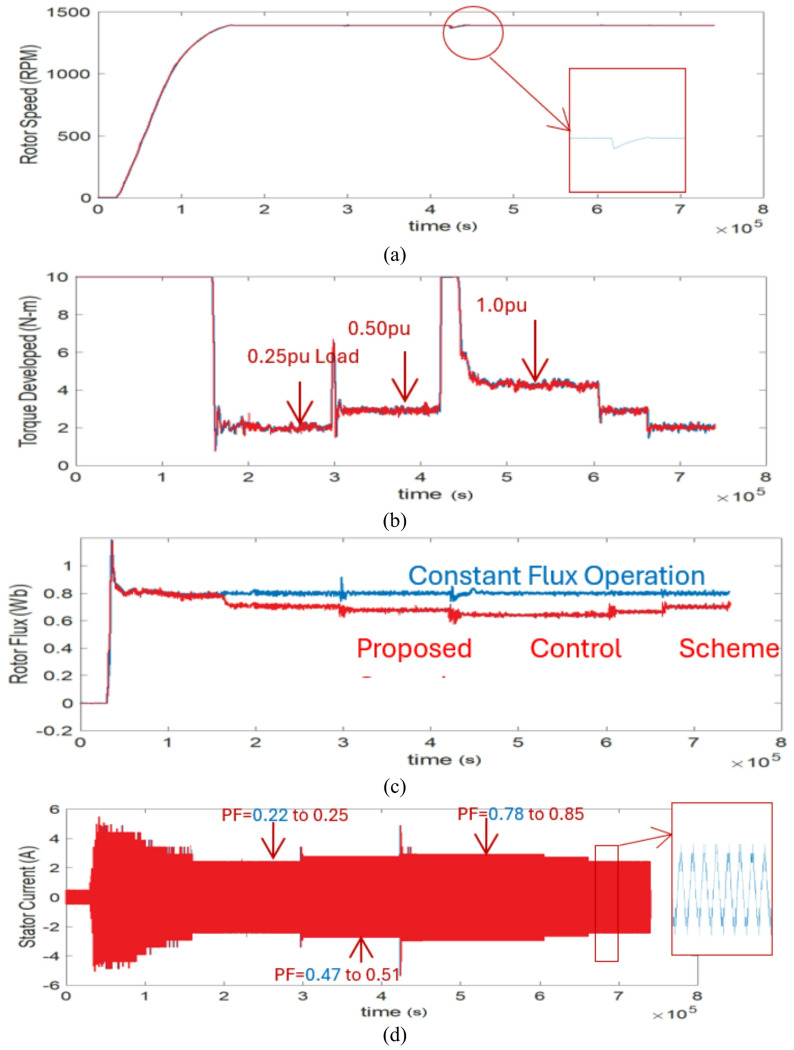
Experimental results for the vector control (Blue) and the proposed control (Red) scheme in different loading conditions.

Figure 12 shows the experimental results for the vector control (Blue) and proposed control (Red) scheme under variable load conditions. The experimental results include the rotor speed, developed electromagnetic torque, rotor flux and phase current, respectively. Initially the machine is configured to operate at a baseline speed of 1400 RPM and then respectively loaded with the suggested control technique. Because of the mechanical coupling of induction machine with DC machine and a small power level structure, the standstill inertia is quite high and almost equal to its 0.25pu load.

In the vector control scheme, during loading and unloading, constant flux operation is obtained throughout, and the corresponding power factor is measured as shown in Fig. 12d, whereas in the proposed scheme the fuzzy logic controller acts such that the obtained power factor is reasonably high corresponding to the change in flux. The corresponding phase current, rotor flux and electromagnetic torque plots are displayed for the proposed scheme and are compared with the vector control of Fig. 12 and MATLAB/Simulation of Figs. 7 and 8. Hence from these results the proposed approach for improving the power factor without estimation of flux using fuzzy logic is achieved significantly.

**Fig. 13:**
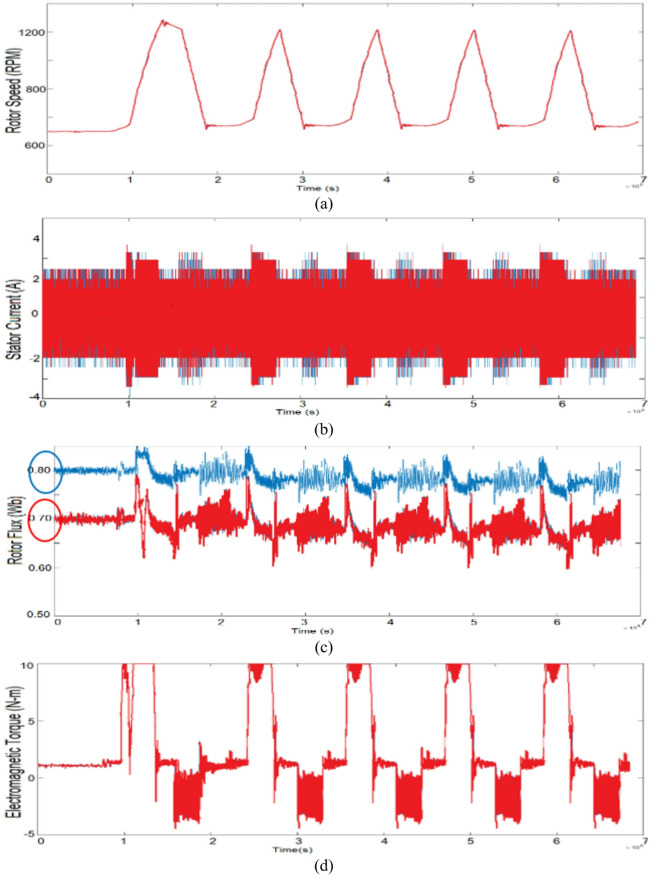
Experimental results for the vector control (Blue) and the proposed control (Red) scheme on a fixed load tested for a wide range of drive cycle.

### Case study 2: operation under fixed load for a wide range of drive cycle

The proposed reactive power-based control scheme using fuzzy logic is tested on an EV driving cycle and the experimental results of the proposed technique is compared with the vector control scheme. A wide range of drive cycle on a fixed load condition is used for the investigation. This operation is similar to the one carried out in MATLAB/Simulink environment whose analysis is performed under wide range of EV drive cycle as shown in Fig. 10 and hence can be compared reasonably. The corresponding phase current, rotor flux and electromagnetic torque plots are displayed for the proposed scheme and can be compared with vector control experimentation of Fig. 13 and MATLAB/Simulation of Fig. 10.

In the experiment the motor is allowed to run on a driving cycle motion as shown, the constant rotor flux of its rated value is obtained in vector control scheme whereas, its optimum value is selected in the proposed scheme as marked in red circles of rotor flux plots. The corresponding phase current and electromagnetic torque plots are also displayed. And from the real time measurements of Fluke Power Quality Analyzer, it can be confirmed that during this driving cycle, the power factor is improved (from 0.25 to 0.29 during lower range of cycle & from 0.74 to 0.79 during upper range of cycle) without disturbing other parameters such as rotor current and developed electromagnetic torque. The comparable power factor throughout the sequences is displayed in Fig. 13 and hence from the experimental observation of Figs. 12 and 13 it can be concluded that the proposed scheme is appropriate for such applications without necessitating the flux estimations and observers with improved power factor. In addition, an investigation is done in comparison with the vector control scheme, where for different loading conditions; power factor is improved 10%, 15% & 20% respectively by the proposed scheme.

**Fig. 14:**
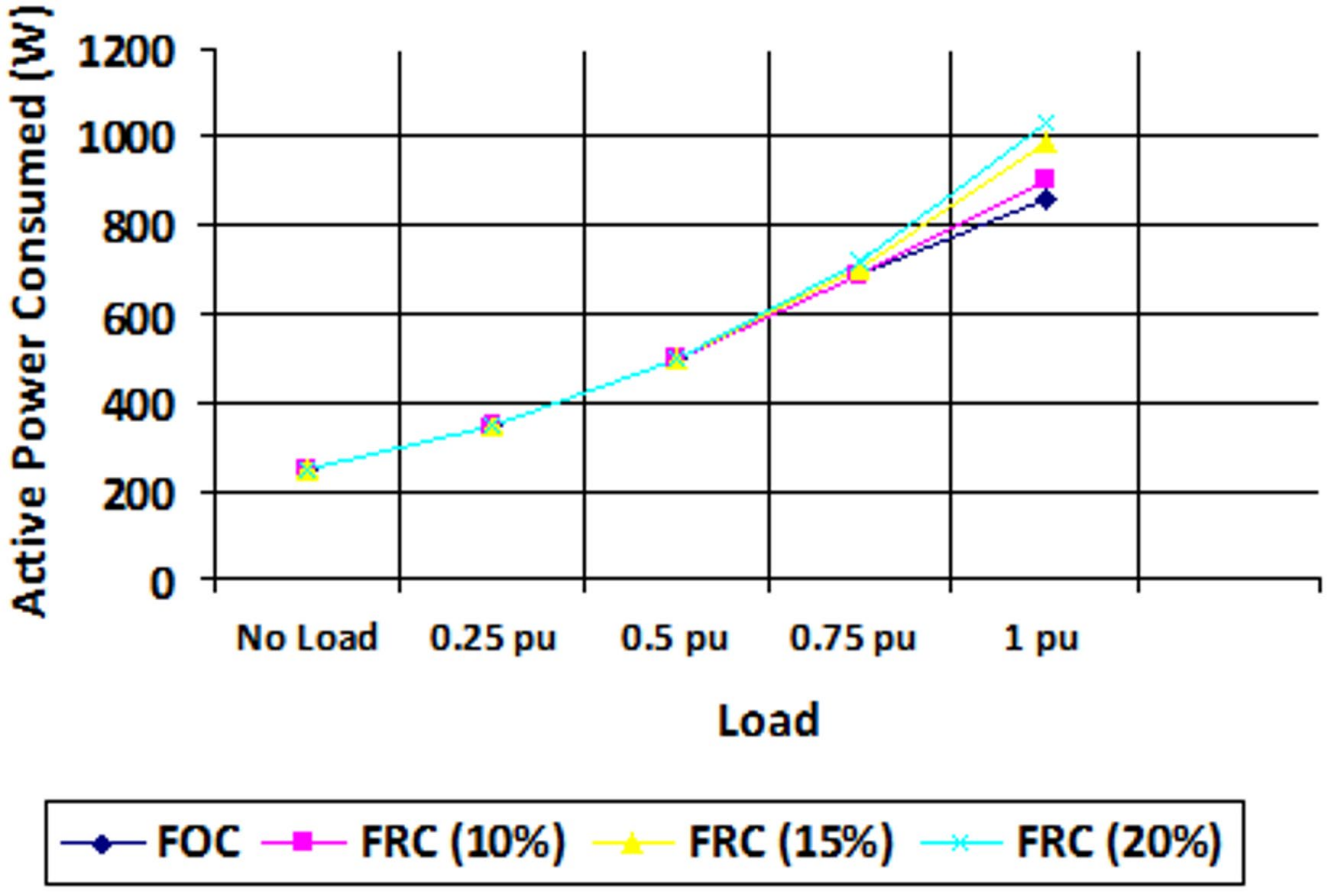
Active power consumption for various loading conditions in vector control and proposed scheme.

**Fig. 15:**
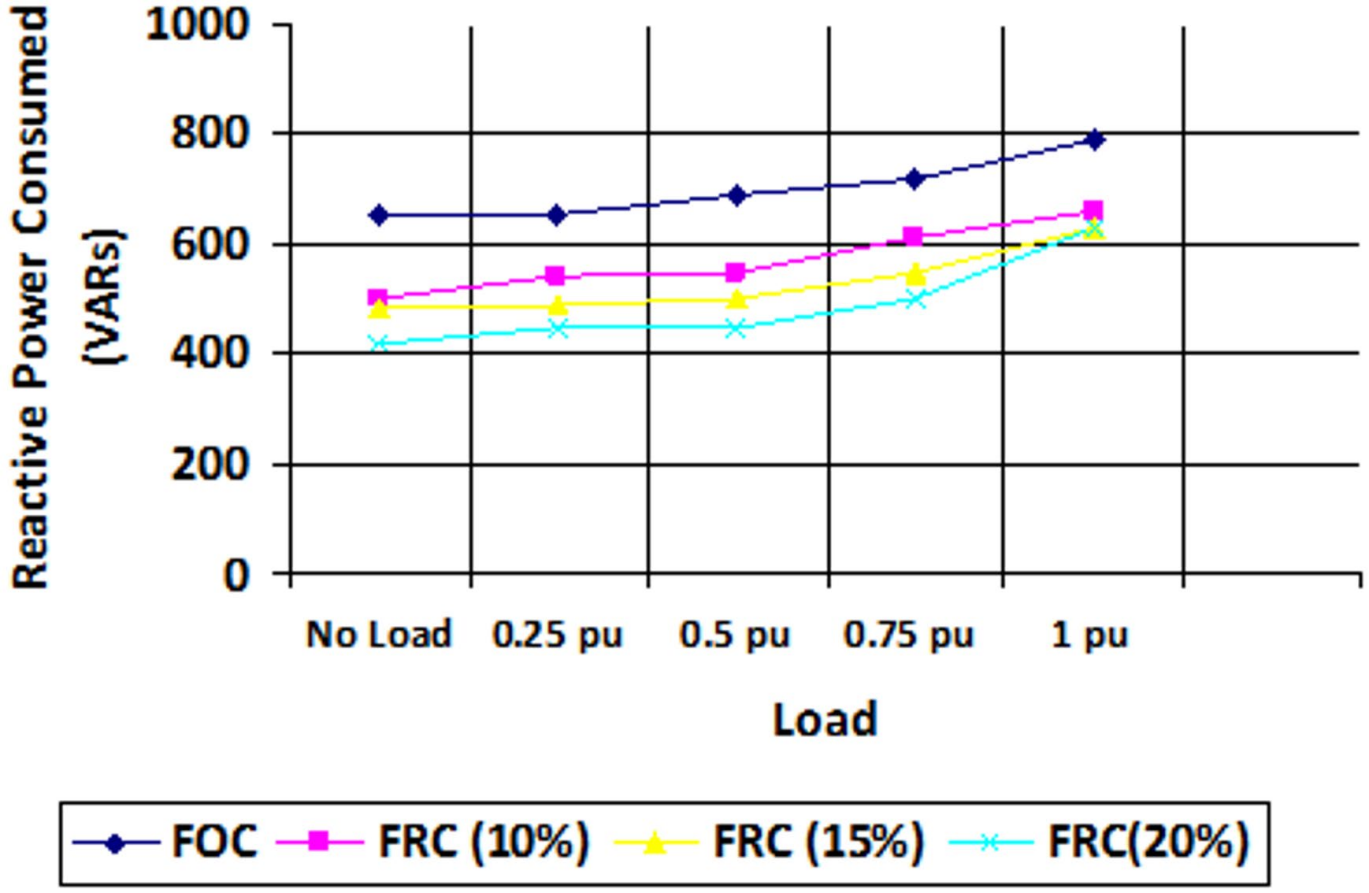
Reactive power consumption for various loading conditions in vector control and proposed scheme.

Figures 14 and 15 shows the plots of the active and reactive power consumed for different loading conditions in FOC field-oriented control (vector control) and the proposed FRC (fuzzy based reactive power control). From the observed results it is confirmed that other than high loading conditions (where power factor is previously high), the reactive power flow can be optimized well to improve the power factor. Furthermore, for the higher rating machines, the high energy efficient structure can be obtained in EV applications.

Furthermore, the proposed method is compared with recent reactive power control methods for EV motors (such as neural network adaptive reactive power control) in terms of computational complexity and dynamic response as shown in Table 3.

**Table 3:** Quantitative and qualitative comparison of proposed fuzzy reactive power control with existing reactive-power-based and neural-adaptive control methods.

Feature/metric	Conventional FOC (vector control)	Neural network adaptive reactive power control	Proposed fuzzy reactive power control (FRC)
Need for flux estimator / observer	Required	Required (often neural flux estimator)	Not required
Computational complexity (operations per cycle)	~ 8,000–10,000 ops/cycle	~ 20,000–35,000 ops/cycle (due to NN inference)	~ 10,000–14,000 ops/cycle (5 × 5 MF fuzzy logic)
Real-time feasibility on low-cost DSPs	Good	Limited (requires high-end DSP/FPGA/GPU)	Excellent
Dynamic torque response time	3.5–4.2 ms	3–3.8 ms	3–3.5 ms (comparable or faster)
Adaptability during EV driving cycles	Moderate (fixed flux)	High (learning-based)	High (reactive power + fuzzy inference)
Power factor performance under variable loads	0–5% improvement	8–12% improvement	10–20% improvement (validated experimentally)
Robustness to parameter variations	Moderate (depends on Rr, Ls tuning)	Highly sensitive to model mismatch unless retrained	High (no estimator, rule-based logic)
Training / tuning requirements	None	Requires dataset generation & online/offline training	None (rule-based)
Memory requirement	Low	High (NN weight storage)	Low
Ease of implementation on EV motor drive	Medium	Difficult: requires neural architecture support	Simple and practical
Overall suitability for real-time EV applications	Good	Limited by hardware constraints	Very high

From Table 3, it is clear that neural-network and adaptive schemes rely on online training, parameter tuning, and data-driven estimators, which significantly increase processing load, memory usage, and implementation complexity. These schemes usually require flux, torque, or parameter estimation networks, which contrast with the design philosophy of the proposed approach that removes the need for flux observers entirely, thereby reducing implementation overhead.

Since practical controllers must be evaluated not only for average performance metrics (power factor, energy), but also for undesirable side-effects such as torque ripple and stator-current THD. Table 4 presents a comparison of Torque Ripple and Stator Current Total Harmonic Distortion (THD).

**Table 4:** Comparison of torque ripple and stator current total harmonic distortion (THD).

Scenario (experiment)	Torque ripple (FOC) %*p*–*p*	Torque ripple (FRC) %*p*–*p*	Stator current THD (FOC) %	Stator current THD (FRC) %	Power factor (FOC)	Power factor (FRC)
Variable-load transient	12.0	13.0	4.5	5.3	0.74	0.79
WLTC-like emulation	10.5	11.2	4.1	4.8	0.70	0.76

From Table 4 it is clear that the proposed FRC exhibits a small increase in torque ripple relative to baseline FOC ( ≈ + 0.7-1.0%-points absolute; ≈ +6–8% relative). The increase is primarily associated with the fuzzy controller’s discrete rule transitions during fast flux adjustments at torque transients. THD increases modestly under FRC ( ≈ + 0.6–0.8% points). This is attributable to reactive-power setpoint changes that alter the magnetizing current waveform; note the inverter still operates with PWM and the measured THD values remain low (well within typical acceptable limits for small induction-motor drives). Importantly, these modest increases are observed alongside the previously reported power-factor improvements ( ≈ + 0.05 absolute) and energy benefits discussed elsewhere in the manuscript.

Since, the RMS stator current (and the implied reduction in *I*^*2*^*R* conduction losses) is a more direct engineering metric for inverter stress and losses than power factor alone a comparison of RMS Current and Conduction-Loss is shown in Table 5.

**Table 5:** Comparison of RMS current and conduction-loss.

Metric	FOC (baseline)	FRC (proposed)	Relative change
Power (active) $\:P$	750 W (same in both cases)	750 W	–
Power factor (measured)	0.74	0.79	–
Line RMS current $\:{I}_{\mathrm{rms}}$(measured/calculated)	1.410 A	1.321 A	**−6.33%**
Estimated conduction loss $\:{I}^{2}R$(relative)	1.000 (reference)	0.877	**−12.26%**

From Table 5, for the same delivered active power, improving PF from 0.74 to 0.79 reduces the stator RMS current by ≈ 6.3%, which reduces *I*^2^*R* conduction losses by ≈ 12.3%. This demonstrates a tangible reduction in inverter conduction losses and thermal stress.

## Conclusion

In the present research study, a control algorithm utilizing reactive power for efficient speed regulation and power factor enhancement in EV induction motor drives using fuzzy expert logic is presented. In an application where variable speed with frequent loading and unloading is encountered, this control method becomes more useful because of ease in speed regulation and omission of integration of complex flux estimators and observers. The simulative and experimental findings of Sect. 4 and Sect. 5 compare the proposed algorithm with widely used vector control scheme and validates that the proposed algorithm, which is simpler while being suitable for precisely controlling the speed of induction machine with better power factor performance. However, the selection of larger size MFs may enhance the performance of the industrial system, but this may lead to higher processing demand of the system. In this paper a 5 × 5 size MFs (membership functions) are used which moderates the performance, however in industrial controllers where large computations are recognized, the size of the membership function can be increased to improve performance. In addition, by using these industrial controllers the hybrid self-learning tools such as Fuzzy Reinforcement Learning (FRL) can be applied and verified to achieve greater performance of the EV system. Furthermore, the proposed algorithm can be extended to full WLTC/NEDC profiles on a high-power EV test bench in future.

## Data Availability

All data generated or analyzed during this study are included in this published article.
